# Modulation of cardiac resident macrophages immunometabolism upon high-fat-diet feeding in mice

**DOI:** 10.3389/fimmu.2024.1371477

**Published:** 2024-06-28

**Authors:** Simeng Zhu, Yujia Liu, Guofang Xia, Xiaoqing Wang, Ailian Du, Jin Wu, Yanpeng Wang, Yuanlong Wang, Chengxing Shen, Peng Wei, Congfeng Xu

**Affiliations:** ^1^ Department of Cardiology, Sixth People’s Hospital, Shanghai Jiaotong University School of Medicine, Shanghai, China; ^2^ Tongren Hospital, Shanghai Jiaotong University School of Medicine, Shanghai, China; ^3^ Xinhua Hospital, Shanghai Jiaotong University School of Medicine, Shanghai, China

**Keywords:** cardiac resident macrophages, RNA-seq, high-fat diet, inflammation, cellular compartment

## Abstract

**Background:**

A high-fat diet (HFD) contributes to various metabolic disorders and obesity, which are major contributors to cardiovascular disease. As an essential regulator for heart homeostasis, cardiac resident macrophages may go awry and contribute to cardiac pathophysiology upon HFD. Thus, to better understand how HFD induced cardiac dysfunction, this study intends to explore the transcriptional and functional changes in cardiac resident macrophages of HFD mice.

**Methods:**

C57BL/6J female mice that were 6 weeks old were fed with HFD or normal chow diet (NCD) for 16 weeks. After an evaluation of cardiac functions by echocardiography, mouse hearts were harvested and cardiac resident CCR2^-^ macrophages were sorted, followed by Smart sequencing. Bioinformatics analysis including GO, KEGG, and GSEA analyses were employed to elucidate transcriptional and functional changes.

**Results:**

Hyperlipidemia and obesity were observed easily upon HFD. The mouse hearts also displayed more severe fibrosis and diastolic dysfunction in HFD mice. Smart sequencing and functional analysis revealed metabolic dysfunctions, especially lipid-related genes and pathways. Besides this, antigen-presentation-related gene such as *Ctsf* and inflammation, particularly for NF-κB signaling and complement cascades, underwent drastic changes in cardiac resident macrophages. GO cellular compartment analysis was also performed and showed specific organelle enrichment trends of the involved genes.

**Conclusion:**

Dysregulated metabolism intertwines with inflammation in cardiac resident macrophages upon HFD feeding in mice, and further research on crosstalk among organelles could shed more light on potential mechanisms.

## 1 Introduction

The obesity ratio has increased remarkably in China over the past four decades, and 16.4% adults were obese in 2015–2019, according to Chinese criteria (1). The growing burden of obesity could be driven by lack of physical activity and alterations in dietary patterns, including increased high-fat and high-sugar consumption. A high-fat diet (HFD) usually results in hyperlipidemia, hyperglycemia, and insulin resistance and increases the secretion of chemokines, cytokines, and adipokines, inducing oxidative stress, inflammation, cell apoptosis, and impairment of metabolic hemostasis (2–4). Thus, high-fat diet and obesity bring a series of health threats. Epidemiological evidence suggest a strong relationship between obesity and cancer (5), metabolic syndrome (6), neurodegeneration (7), and cardiovascular disease (CVD) (8). As a major cause of CVD, obesity accounts for 65%–75% of risk for primary hypertension (9) and increases the risks of heart failure and myocardial infarction by 5%–7% (10) and 12% (11), respectively.

The systematic inflammation linked obesity with cardiac dysfunction and adverse cardiac remodeling. In this process, macrophages, a major component of the innate immune system, play an essential part. At first, macrophages were believed to be originated from monocytes (12), while tissue-resident macrophages were described lately, which were maintained independently of circulating monocytes (13). Further research confirmed that tissue-resident macrophages are derived from embryonic progenitors in the yolk sac and keep their self-renewing capacity after birth (14). Aside from their common ability to phagocytose, tissue-resident macrophages also play a critical role during development and regulate organ homeostasis in adulthood (15). Furthermore, tissue-resident macrophages possess tissue-specific functions—for example, microglia help shape neural circuits during development (16), Kupffer cells participate in lipid and iron metabolism (17), and alveolar macrophages maintain surfactant homeostasis (18).

Cardiac resident macrophages existed in a similar environment with microglia, both surrounded by non-self-renewal cells. As the most abundant immune cells in the heart (19), cardiac macrophages could be primarily identified by CX3CR1 (C-X3-C motif chemokine receptor 1) expression (20). Furthermore, cardiac macrophages could be divided into embryonic resident macrophages and hematogenic recruited macrophages by the expression of CCR2 (C-C chemokine receptor 2), an important chemokine receptor for cell migration (21). CCR2^-^ resident macrophages derive from embryonic origin, help in angiogenesis and cardiomyocyte proliferation after injury (22), participate in electrical conduction (23), and mediate metabolic stability (24). However, the dysfunction of cardiac resident macrophages in hyperlipidemia status and its potential role in obesity-associated cardiovascular disease remains under debate.

Thus, in the current study, we used an innovative tool, Smart (switching mechanism at the 5′ end of the RNA transcript) RNA sequencing, to better define the transcriptional and functional changes in cardiac resident macrophages of hyperlipidemic mice. Furthermore, we tried to identify the potential link between inflammation and metabolism in cardiac macrophages.

## 2 Methods

### 2.1 Animals and animal model

All animal experiments were approved by the Institute of Animal Care and Use Committees (IACUC) at Shanghai Model Organism (2022–0015). All of the mice were kept in a specific pathogen-free condition with a 12-h/12-h light–dark cycle under a room temperature of 24°C. All of the mice had access to food and water *ad libitum*.

To establish HFD models, 6-week-old C57BL/6J female mice were randomly assigned to a HFD (60% kcal from fat, 20% protein, and 20% carbohydrate) or normal chow diet (NCD) (10% kcal fat, 20% protein, and 70% carbohydrate) group. Seven HFD mice and seven NCD mice were fed for 16 weeks. At the end of the experiment, blood was collected from the orbital plexus after a 12-h fasting period, and hearts were excised and weighted after the mice were sacrificed. Among them, three mice from each group were sacrificed for Smart RNA sequencing, and the others were prepared for phenotypic experiments. During this period, the mice were weighed every week, and physical examination was performed to check their health condition.

### 2.2 Lipid profile

Mouse fasting serum triglyceride, total cholesterol, low-density lipoprotein cholesterol (LDL-c), and high-density lipoprotein cholesterol (HDL-c) were measured in the central lab of Tongren Hospital (Aptio™ automation, Siemens, Germany).

### 2.3 Echocardiography

The mice were anesthetized with 2% isoflurane after shaving the chest. The cardiac function and geometry were evaluated using two-dimensional and M-mode echography (Vevo 3100, VisualSonics, Fujifilm, Toronto, ON, Canada) with an MS-400 imaging transducer. Two-dimensional (2D) images were acquired in the apical four-chamber view, with a consistent maintenance of the heart rate at 400–450 beats per minute (bpm). Key cardiac parameters, including the ratio of the peak early filling velocity (E-wave) to the peak atrial contraction velocity (A-wave) across the mitral valve (E/A) and isovolumetric relaxation time (IVRT), were precisely documented in apical four-chamber view. To ensure accuracy, all measurements represent the average values obtained over three consecutive cardiac cycles.

### 2.4 Histological examination

After euthanization, the mice’s hearts were harvested and immersed in 4% neutral-buffered formalin at room temperature for 24 h. Subsequently, the specimens underwent embedding in paraffin, followed by sectioning at 4-μm thickness. Then, the slides were subjected to hematoxylin–eosin staining on paraffin-embedded sections to evaluate cardiac inflammation, and Masson’s trichrome staining was employed to assess fibrosis.

### 2.5 Detection of cytokines in heart tissue

The harvested heart tissue was cut into small pieces and homogenized, and the supernatant was collected for ELISA (R&D, USA) assay to detect the levels of IL-1β, IL-6, TNF-α, and IFN-γ.

### 2.6 Adult mouse heart single-cell suspension generation and CCR2^-^ macrophage sorting

The mice’s hearts were harvested, and the left ventricle tissue was finely minced into approximately 1-mm^3^ pieces using delicate scissors and digested in PBS containing 450 U/mL type I collagenase, 450 U/mL type II collagenase, 150 U/mL type IV collagenase (all from Worthington Biochemical Corporation, Lakewood, NJ, USA), and 60 U/mL DNase I for 60 min at 37°C with gentle agitation. Following digestion, the hearts underwent repeated aspiration with a pipette and passed through a 70-μm cell strainer to prepare single-cell suspensions. Furthermore, the cells were incubated with anti-CD16/32 antibody (clone# 93, eBioscience) for 15 min and stained at 4°C for 30 min with the following antibodies: anti-CX3CR1-PE-cy7 (clone# SA011F11, BioLegend), anti-CD11b-BV605 (clone# M1/70, BD Bioscience), and anti-CCR2-BV421 (clone# SA203G11, BioLegend). All labeled cells were sorted using FACSAria™ III Cell Sorter (BD Biosciences, USA), and after appropriate gating, a minimum of 5,000 cardiac resident macrophages were sorted for further sequencing. The data were analyzed using FlowJo software (TreeStar Inc., USA).

### 2.7 Smart-seq of FACS-isolated RCMs

Total RNA was isolated from sorted resident macrophages using TRI Reagent (Sigma Aldrich, USA). Sequencing libraries were generated utilizing the Smart-Seq v4 Ultra Low Input RNA Kit from Clontech (Japan). The library preparation process encompassed the following steps: (1) construction of cDNA library, (2) cDNA size selection and purification (150–300 bp), (3) creation of a sequencing-ready library (via incorporation of i5 and i7 adaptors by PCR method), (4) evaluation of library quality (by examining its size and concentration through Agilent 2100 Bioanalyzer), and (5) sequencing of the library conducted on Illumina platforms employing a 2 × 150-bp paired-end sequencing protocol. Raw data quality assessment was conducted using FastQC, and Cutadapt was used to trim poor-quality sequences and adaptors. Further analysis was performed with R (v4.04). For differential expression analysis, package DESeq2 (v 3.18) was utilized with a log_2_ (fold change) threshold set at >2 or <-2 and a false discovery rate (FDR) <0.05. Principal component analysis (PCA) was performed using the plotPCA function in DESeq2. Heatmaps and volcano plot of differentially expressed genes (DEGs) were generated by hierarchical clustering using the hclust function in package stats (v 3.6.2). Gene Ontology (GO), Kyoto Encyclopedia of Genes and Genomes (KEGG) analysis, and Gene Set Enrichment Analysis (GSEA) were conducted by clusterProfiler (v 4.10.0). All of the figures were plotted using ggplot2 (v 3.4.2).

### 2.8 Quantitative reverse-transcription PCR

The mice’s hearts were harvested as described above. The harvested hearts were cut into small pieces, and tissue RNA was extracted using TRI reagent (Sigma Aldrich). RNA was reverse-transcribed to cDNA with HiScript II Reverse Transcriptase (Vazyme, Jiangsu, China). Next, we performed RT-PCR using a SYBR qPCR Master Mix (Vazyme), and the primer sequence is shown in **Supplementary Table S1**. 2^-ΔΔCt^ method was applied to calculate the relative gene expression levels normalized to GAPDH.

### 2.9 Statistical analysis

Data were analyzed using Graphpad Prism (v 9.5). Differences between unpaired data were evaluated with a two-tailed Student’s *t*-test. The difference was considered as statistically significant if *P <*0.05.

## 3 Results

### 3.1 HFD-fed dyslipidemic mice displayed cardiac dysfunction

**Figure 1:**
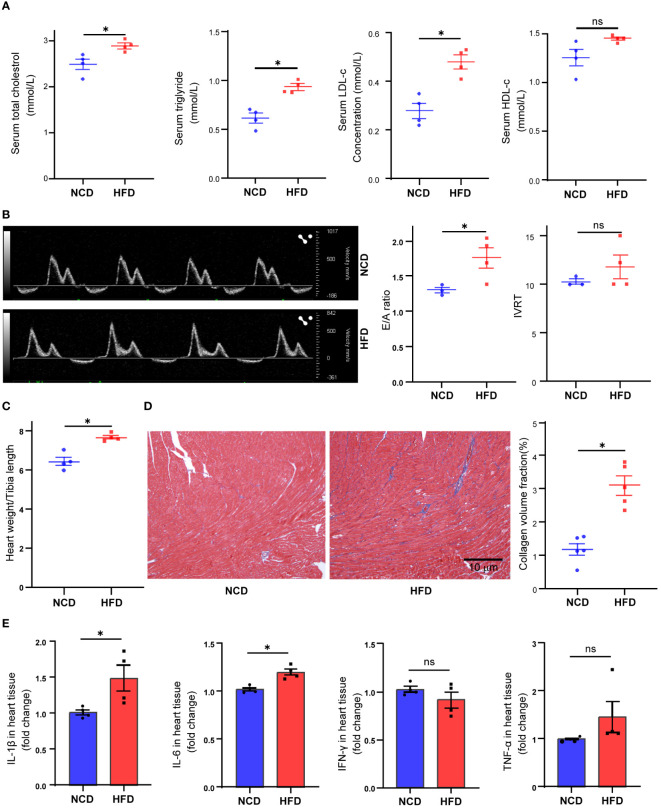
Metabolic profiles and cardiac functional alteration in high-fat diet (HFD) mice. Serum total cholesterol, triglyceride, low-density lipoprotein cholesterol, and high-density lipoprotein cholesterol levels in mice treated with normal chow diet (NCD) or HFD **(A)**. Cardiac diastolic function of NCD and HFD mice evaluated by the ratio of early to late ventricular filling velocities (E/A ratio) and isovolumetric relaxation time (IVRT) **(B)**. Overall heart weight/tibia length ratio in NCD and HFD mice **(C)**. Masson trichrome staining of sections from NCD and HFD heart tissues **(D)**. Cytokine levels in heart tissue from NCD and HFD heart tissues **(E)**. Data are shown as mean ± SEM. *P < 0.05. NS, No Significance. All statistical analyses were performed using two-tailed Student’s *t*-test.

In the present study, we fed mice with HFD for 16 weeks. At the end of the treatment, the HFD-fed mice showed a significant increase in body weight (37.15 vs. 26. 78 g, *P* < 0.001, data not shown), serum total cholesterol concentration (*P* = 0.022), triglyceride concentration (*P* = 0.002), and LDL-c concentration (*P* = 0.003) (**Figure 1A**). To explore the effect of HFD on mouse heart, we further compared the difference of cardiac function in two groups. With the help of pulse-wave doppler imaging, we observed that mice fed a high-fat diet exhibited reduced E/A ratios (**Figure 1B**), suggesting that high fat may induce diastolic dysfunction. However, we observed no significant difference in left ventricular systolic function between the two groups (**Supplementary Figure S1**). Following the heart harvest, we found that heart weight/tibia length was remarkably elevated in HFD-fed mice (**Figure 1C**), indicating concentric cardiac hypertrophy. Also, Masson trichrome staining identified hyper-fibrosis in high-fat heart (**Figure 1D**), potentially providing insights into the underlying cause of diastolic dysfunction. As high fat and obesity potentially drive inflammation activation (25), so we detected the cytokine levels in HFD-fed mice’s hearts and found that IL-1β and IL-6 production was elevated in HFD-fed mice’s hearts (**Figure 1E**), suggesting inflammation as a mediator in HFD-induced cardiac dysfunction.

### 3.2 Sorting of cardiac resident macrophages

**Figure 2:**
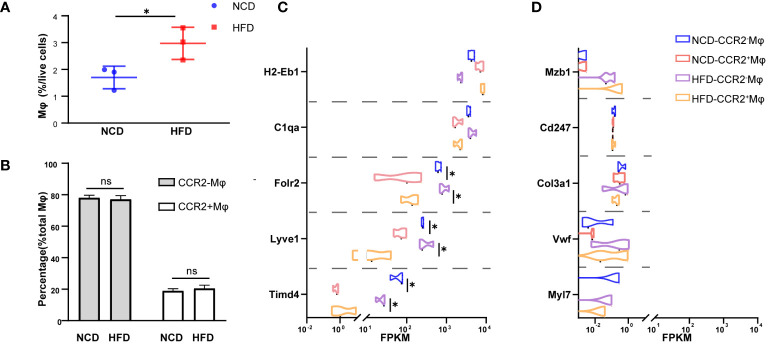
CCR2^-^ cardiac resident macrophage sorting. Percentage of macrophages among living cell **(A)** and CCR2^-^ or CCR2^+^ macrophage among total macrophages **(B)** in normal chow diet (NCD) and high-fat diet (HFD) heart. Expression of macrophage markers of sorted macrophages **(C)**. Expression of other cardiac cell markers of sorted macrophages in NCD and HFD mice **(D)**. Data are shown as mean ± SEM. *P < 0.05. NS, No Significance. All statistical analyses were performed using two-tailed Student’s *t*-test. FPKM, fragments per kilobase of transcript per million fragments mapped.

In the physiological condition, macrophages are the most abundant immune cells in the heart, and most of them are resident macrophages, also known as cardiac macrophages (26). To evaluate the composition in the heart of HFD mice, we sorted cardiac macrophages (CD11b^+^CX3CR^+^) and further distinguished resident and recruited macrophages based on the expression of chemokine receptor CCR2 (**Supplementary Figure S2**). As shown here in **Figure 2A**, macrophages accounted for a larger proportion (2.97% vs. 1.70%, *P* = 0.040) among total cardiac cells in HFD mice compared with the control, while the ratio of CCR2^-^ (embryonic-derived resident) to CCR2^+^ (hematopoietic recruited) cells among macrophages showed no significant difference between the two groups (HFD: 77.01% vs. NCD: 78.05%, *P* = 0.65, in CCR2^-^ macrophages; HFD: 20.47% vs. NCD: 18.93%, *P* = 0.47, in CCR2^-^ macrophages, **Figure 2B**). Next, we had CCR2^+^ and CCR2^-^ macrophages subjected to Smart RNA sequencing. The RNA sequencing results showed that both CCR2^-^ and CCR2^+^ macrophages expressed common macrophage markers, such as *C1qa*, and CCR2- macrophages highly expressed TLF markers (*Timd4*, *Lyve1*, and *Folr2*), which are functional markers of cardiac resident macrophages (**Figure 2C**). While neither of CCR2^+^ nor CCR2^-^ macrophages expressed *Mzb1* (B cell markers), *Cd247* (T cell marker), *Col3a1* (fibroblast markers), *Vwf* (endothelial cell markers), and *Myl7* (cardiomyocyte markers), validating the accuracy of our sorting strategy (**Figure 2D**).

### 3.3 Gene expression alteration of cardiac resident macrophages

**Figure 3:**
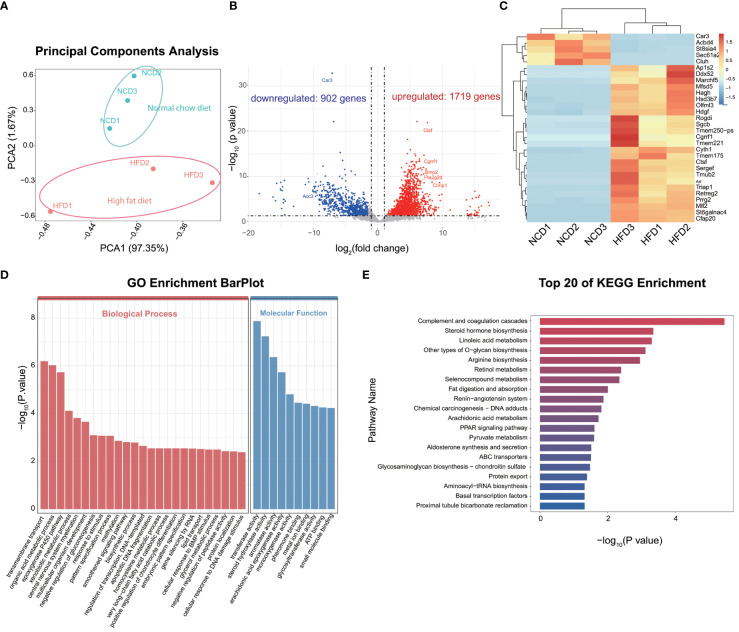
Gene expression profile of CCR2^-^ cardiac resident macrophages revealed by Smart sequencing analysis. Principal component analysis of sequencing for normal chow diet (NCD) and high-fat diet (HFD) CCR2^-^ cardiac macrophages **(A)**. Volcano plot **(B)** and heatmap **(C)** showing the top differentially expressed genes (DEGs) between NCD and HFD groups; red signifies upregulated and blue for downregulated genes. Gene ontology (GO) **(D)** and Kyoto Encyclopedia of Genes and Genomes (KEGG) **(E)** analysis showing the top enrichment pathways of DEGs.

To gain insights into the effect of HFD on cardiac resident macrophages, we focused on the gene expression profile of CCR2^-^ macrophages, which ontogenically are mainly embryonic-derived and are essential for cardiac homeostasis (27). PCA distinguished HFD cardiac macrophages from the control group (**Figure 3A**). The analysis of DEGs revealed 1,719 significantly upregulated genes and 902 downregulated genes, with *Car3* (*carbonic anhydrase 3*), *Abcd4*, and *Cluh* being the top downregulated genes, while *Ap1s2*, *Marchf5*, and *Ctsf* (*Cathepsin F*) were among the top upregulated genes (**Figures 3B, C**). Among them, four (*Abcd4*, *Cluh, Ap1s2*, and *Marchf*5) were involved in organelle regulation or communication (*Cluh* and *Marchf5* for mitochondrion biogenesis and fission, respectively; *Ap1s2* for protein sorting in trans-Golgi network and endosome; and *Abcd4* for vitamin B12 transport from lysosome), which prompts us to explore organelle-specific changes (see Section 3.6), and two of them are enzymes (see the discussion text for details). GO analysis identified that DEGs were enriched in metabolism pathways, such as organic acid metabolism, gluconeogenesis regulation, very-long chain fatty acid catabolic, glycerol metabolism, or lipid transport (**Figure 3D**). In order to further understand the biological processes induced by high-fat diet, we made a KEGG enrichment analysis to reveal the signaling pathways, and we found that signaling pathways associated with complement activation, inflammation, and lipid metabolism were on top of the candidates. Our results accentuate that metabolism and inflammation were potential targets induced by HFD (**Figure 3E**) in cardiac resident macrophages. We also detected the gene alterations in CCR2^+^ macrophages, and lipid metabolism and inflammation-related pathways were also noted in KEGG and GO enrichment analysis (**Supplementary Figure S3**). As embryogenic macrophages are CCR2^-^ and make up most cardiac resident macrophages, we thus further investigated the CCR2^-^ macrophages.

### 3.4 Metabolic changes in cardiac resident macrophages induced by HFD feeding

**Figure 4:**
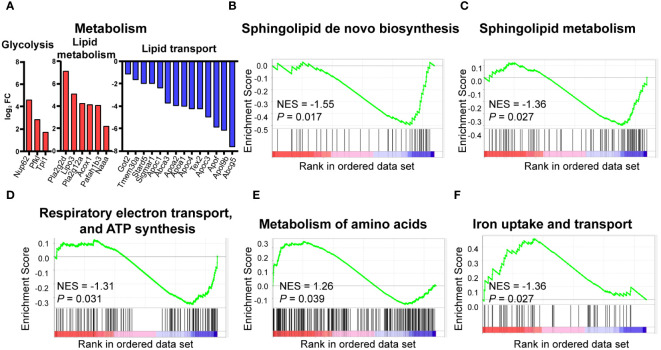
Metabolic pattern shifts of CCR2^-^ macrophages upon high fat stress. Bar plot showing the dysregulation of the indicated metabolic genes in cardiac CCR2^-^ macrophages of the high-fat-diet group **(A)**. Gene set enrichment analysis of metabolism-related gene sets for sphingolipid metabolism **(B)**, sphingolipid *de novo* biosynthesis **(C)**, respiratory electron transport, ATP synthesis by chemiosmotic coupling, and heat production by uncoupling proteins **(D)**, metabolism of amino acid and derivates **(E)**, and iron uptake and transport **(F)**.

As HFD considerably affects the signaling pathways of metabolism, we further analyzed the metabolism-associated DEGs. Three DEGs are related to glycolysis, including *Nup62*, *Pfkl*, and *Tpi1*. These genes were upregulated in HFD cardiac macrophages compared to the control group. Similarly, genes involved in lipid metabolism, such as *Pla2g2d*, *Lipo3*, and *Naaa*, were also significantly upregulated. However, lipid-transport-related genes, including *Abcg5*, *Apo3*, and *Stard5*, showed a significant downregulation, suggesting that lipid transport was dysregulated in HFD-fed mice (**Figure 4A**). GSEA analysis also suggested that pathways associated with lipid metabolism (sphingolipid metabolism, **Figure 4B**), sphingolipid *de novo* biosynthesis **Figure 4C**), energy metabolism (respiratory electron transport, ATP synthesis by chemiosmotic coupling, and heat production by uncoupling proteins, **Figure 4D**), amino acid metabolism (metabolism of amino acid and derivates, **Figure 4E**), and trace element metabolism (iron uptake and transport, **Figure 4F**) were statistically upregulated in the HFD group, suggesting the effect of lipid-containing food on metabolic disorder in cardiac resident macrophages, including the downregulation of lipid transport and upregulation of glycolysis and fatty acid synthesis.

### 3.5 HFD contributes to inflammatory state shifting

**Figure 5:**
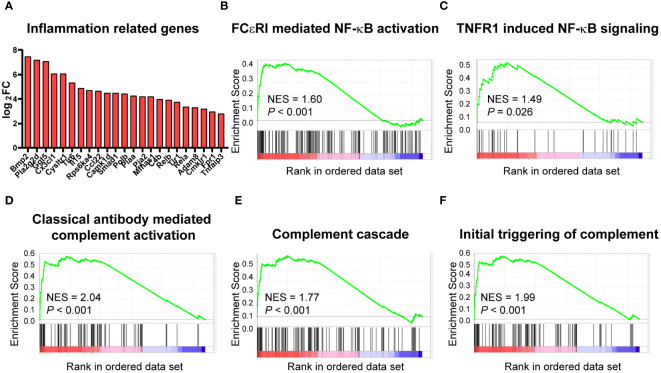
High-fat diet altered the inflammatory status of cardiac CCR2^-^ macrophages. Bar plot showing the upregulation of the indicated inflammation-associated genes in cardiac CCR2^-^ macrophages of the high-fat-diet group **(A)**. Gene set enrichment analysis of inflammation-related gene sets for FCεRI-mediated NF-κB activation **(B)**, TNFR1-induced NF-κB signaling pathway **(C)**, complement activation gene sets for classical antibody-mediated complement activation **(D)**, complement cascade **(E)**, and initial triggering of complement **(F)**.

A tight link has been established between metabolism and inflammation in immune cells, and macrophages are no exception. As mentioned above, cardiac macrophages underwent significant metabolism change upon high-fat diet, so we further analyzed the effect of HFD on inflammation. RNA sequencing analysis showed that many of the DEGs related to inflammation, such as *Bmp2*, *Cx3cl1*, and *Tlr6* were upregulated in the HFD group (**Figure 5A**). This is consistent with the activation of NF-kB signaling cascade [FCεRI mediated NF-κB activation (**Figure 5B**) and TNFR1 induced NF-κB signaling pathway (**Figure 5C**)] based on GSEA. Similarly, the analysis also identified that complement activation was upregulated after HFD feeding [NES = 2.04, *P* < 0.001 for classical-antibody-mediated complement activation (**Figure 5D**), complement cascade (**Figure 5E**), and initial triggering of complement (**Figure 5F**)]. Complement activation and inflammation-associated pathways were significantly upregulated in HFD macrophages, predicating a shift to pro-inflammatory status.

### 3.6 Organelle-specific localization of critical genes in cardiac resident macrophages

**Figure 6:**
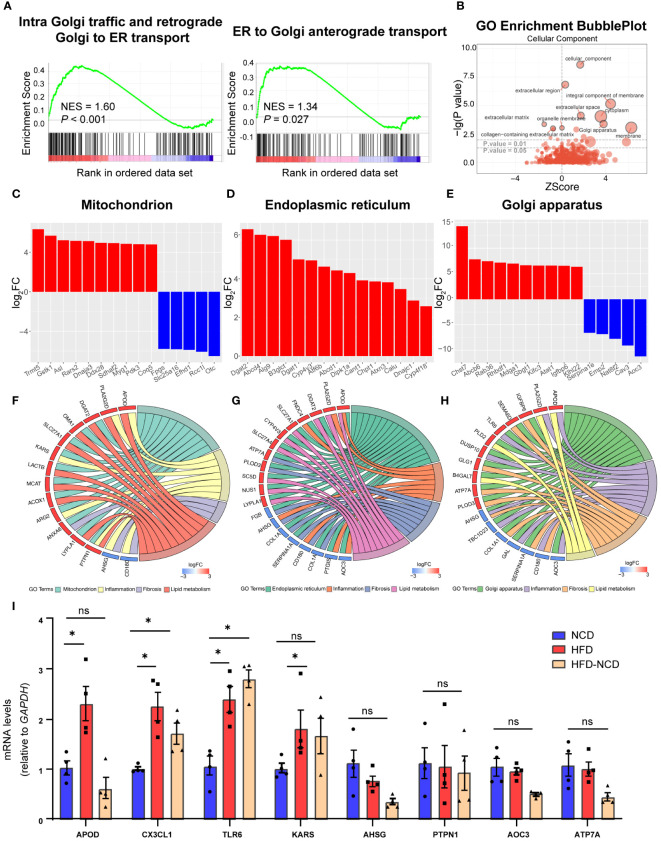
Cellular compartment localization of lipid metabolic and inflammatory genes. Gene set enrichment analysis of intracellular traffic gene sets for intra Golgi traffic and retrograde Golgi to ER transport and ER to Golgi anterograde transport **(A)**. Gene ontology analysis showing the cellular component enrichment of DEGs **(B)**. Bar plot showing the dysregulated mitochondrion **(C)**, Golgi apparatus **(D)**, and endoplasmic reticulum **(E)** localized genes of the high-fat diet (HFD) group; red for upregulated and blue for downregulated. Chord plot showing the overlapped genes among lipid metabolism, inflammation, fibrosis, and different organelles: **(F)** mitochondrion, **(G)** endoplasmic reticulum, and **(H)** Golgi apparatus. Red signifies upregulated and blue for downregulated. RT-PCR quantification of *Apod*, *Cx3cl1*, *Tlr6*, *Kars*, *Ahsg*, *Ptpn1*, and *Atp7a* in normal chow diet (NCD), HFD-fed mice, and mice fed with NCD for 3 months after high-fat-diet feeding (HFD-NCD) **(I)**. *P < 0.05, NS, No significance.

Our GSEA also revealed a significant alteration in intracellular trafficking [intra-Golgi traffic and retrograde Golgi to endoplasmic reticulum (ER) transport and Golgi anterograde transport, **Figure 6A**]. There is an extensive intracellular communication network between mitochondria, ER, and Golgi apparatus bridging metabolism and inflammation in resident macrophages, which are essential during their proper functioning (28). Both enhanced lipid metabolism and inflammation response promote fibrosis (29, 30). So, we investigated the DEGs across different cellular components to enhance our understanding of the potential mechanisms underlying hyperlipidemia and cardiac dysfunction. Based on GO analysis, DEGs were enriched in the extracellular region, membrane, and Golgi apparatus (**Figure 6B**). Here we listed the top 15 DEGs in the mitochondrion, ER, and Golgi apparatus, three of the most well-studied organelles in the mammalian cell. In the mitochondrion, 101 genes were significantly upregulated, including *Tmmt5*, *Gstk1*, and *Asl*, while 20 genes were downregulated, like *Fpgs* (**Figure 6C**); 120 DEGs were upregulated, while 49 were downregulated in Golgi apparatus (**Figure 6D**); and 66 DEGs were identified in ER, and all of them were upregulated (**Figure 6E**). In order to enhance our understanding of their function in cardiac dysfunction induced by HFD, we endeavored to identify the overlapping DEGs associated with lipid metabolism and inflammation in CCR2^-^ macrophages.

As intertwinement between metabolism and inflammation usually leads to fibrosis, which plays an essential role for cardiac function and aging (31), we also include DEGs for fibrosis. Among them, 10 overlapping DEGs were spotted in the mitochondrion, with *Kargs* and *Arg2* identified in the inflammation pathway, *Anxa6* in the fibrosis pathway, and the other six genes in lipid metabolism (**Figure 6F**). Moreover, 15 overlapping DEGs were identified in Golgi apparatus, with *Aoc3*, *Tlr6*, *Tbcld23*, *Gal*, *Ahsg*, and *Dusp10* associating with inflammation and *Apod* and *Pla2g2d* with lipid metabolism (**Figure 6G**). As for ER, 17 overlapping DEGs were found. Among them, four were overlapped with inflammation pathway (*Aoc3*, *Fndc4*, *Apod*, and *Ptgis*), and another six DEGs were also identified in lipid metabolism (**Figure 6H**). The key genes for crosstalk of inflammation and lipid metabolism have been validated by RT-PCR, and here we would like to point out the persistent effect of HFD on *Cx3cl1* and *Tlr6*, even after reversing to normal diet for 3 weeks (**Figure 6I**), suggesting the chronic role of lipid overload on inflammation. These results accentuate the critical role of gene expression localization/compartmentalization for the regulation of biological processes, such as metabolism, inflammation, and fibrosis, and further research on how organelles communicate during HFD for cardiac dysfunction would be interesting.

## 4 Discussion

The state and behavior of cardiac resident macrophages are essential for their diverse functions, both physiologically and pathologically. Dysregulation of metabolic pathways, for instance, in diabetes or induced by unhealthy life style perturbs macrophage homeostasis and contributes to the progression of cardiovascular diseases, such as atherosclerosis, myocardial injury, adverse remodeling, and even heart failure (31). Based on RNA-seq, our study provides a scenario of metabolic change induced by HFD, including metabolism of glucose, lipid, and amino acids. Furthermore, the dysregulated metabolism intertwines with inflammation, which shows a complicated crosstalk between these processes.

HFD is a common risk factor for metabolic disorders and various cardiovascular disorders. Several of the previous studies did not report a heart failure upon short-term high-fat stress in wild-type mice (32, 33), so here in our study, we prolonged the HFD feeding time to16 weeks and observed that HFD-fed mice showed diastolic dysregulation, but without visible heart failure. In our study, we noted there were more macrophages in the cardiac tissue of HFD-fed mice, while the proportion of CCR2^+^ and CCR2^-^ cells remained unchanged, which indicated that both the number of recruited and resident macrophages, respectively, were increased. Conventionally, CCR2^+^ cells were believed to be recruited upon injury and inflammation (34), but the origin and trajectory of the increased CCR2^-^ macrophages remained unclear.

As we mainly focused on embryogenic CCR2^-^macrophages, they were sorted for transcriptional analysis, and *Car3* was identified as a DEG with the biggest fold change. *Car3* is a carbonic anhydrase mainly expressed in skeletal muscles, actively participating in lipid metabolism (35). Several studies indicated that HFD increased *Car3* expression in lipid storage organs, like liver and adipose tissue (36, 37). To our surprise, in the present study, *Car3* exhibited a significant downregulation in cardiac macrophages of HFD mice. This disparity suggests that the role of *Car3* in the heart may extend beyond lipogenesis, which surely is worth further investigating into its involvement in other physiological processes. Our previous study suggested it as a key regulator of CHRN degradation, participating in the development of myasthenia gravis (38). Furthermore, its impact on fibrosis and inflammation should be explored given our results in the animal model. In the present study, we also observed a drastic upregulation for *Ctsf*, a cathepsin critical for MHC II antigen presentation (39), prophesying a possible crosstalk between HFD and immunity given the critical role of macrophages. Recently, CTSF has been shown to link with myelin maintenance and complement cascade in myelin oligodendrocyte glycoprotein antibody-associated disease (40). Cathepsins are lysosomal proteases and susceptible to age-related alterations, while CTSF has also been reported to work as a causal factor for age-related macular degeneration (41). These results indicated the alteration of metabolic and inflammatory status in cardiac resident macrophages; thus, we further focused on the related pathways.

Metabolic dysregulation and inflammation upon high-fat stress contribute to cardiac pathological change and dysfunction, such as diastolic function impairment and ventricular mass increase, which are a common pathology for cardiovascular diseases, such as myocardiopathy, with potentially more severe consequences. In fetal heart, glucose, lactate, and pyruvate are the primary fuels for cardiac macrophages, while shortly after birth, the fuel source experiences a shift from glucose to fatty acids (60–70%) in healthy adult heart (31). It is reported that maternal hyperglycemia affects cardiac development, while there are few studies to explore how metabolic derangement impact cardiac macrophages (42). In the current study, all of the DEGs related to glycolysis, including *Nup62*, *Pfkl*, and *Tpi1*, were upregulated, while genes related to glucose oxidation, like *Pdk3* and *Pdha1*, were downregulated, suggesting that glucose metabolism shifts from oxidation to glycolysis in HFD-fed mice. As for the genes involved in lipid metabolism, *Pla2g2d*, *Lipo3*, and *Naaa* were significantly upregulated, promoting anabolism, while lipid-transport-related genes, such as *Abcg5*, *Apo3*, and *Stard5*, were down-regulated, leading to lipid surplus and diverse metabolic syndromes, such as obesity. Moreover, antigen presenter *Ctsf* and fatty acid transporter *Abcd4* were also on the top of the DEG list. This observation directs our attention toward potential alterations in both metabolism and inflammation in HFD mice.

It is well known that HFD disturbs metabolism and promotes immune response (43). Previous studies suggested that HFD impairs macrophage phagocytosis in the intestine (44) and induces pyroptosis in Kupfer cells (45). It is also known that classically activated macrophages (M1) display enhanced glycolysis, ROS production, and a proinflammatory cytokine profile, while alternatively activated macrophages (M2) to exhibit increased oxidative phosphorylation, arginase 1 expression, and an anti-inflammatory cytokine profile (46). In monocyte-derived macrophages after myocardial infarction, this immunometabolic phenotype has also been observed in M1 and M2 polarization, but not in resident macrophages (47). Given the entangled interaction between metabolic disorders and inflammatory state, it is understandable that there is a bunch of inflammation-associated genes changed in cardiac resident macrophages following HFD. Genes such as *Tlr1*, *Tlr6*, *Irf3*, and *Tyk2* and chemokines like *IL-15* and *IL-27* were considerably upregulated and almost surely boosted the genes pertaining to NF-κB signaling. At the current stage, there are no convincing studies to show how HFD contributes to cardiac macrophage activation. However, it is very likely that fuel overload leads to metabolic derangements in macrophages: increased glycolysis promotes a proinflammatory state through AXL signaling (48); increased glycolysis and the pentose phosphate pathway can activate pyruvate kinase muscle enzyme 2 (PKM2), promoting the transcription of IL-6 and IL-1β (31); and cholesterol crystals can trigger NLRP3 inflammasome activation, leading to the secretion of IL-1β and IL-18 (49). Interestingly, our study identified an upregulation of FcεRI-mediated NF-κB activation (**Figure 5B**), suggesting an essential role of IgE in cardiac resident macrophage activation. Actually, IgE has been shown to promote macrophage polarization to M1 type and contribute to foam cell formation, putatively through FcεRI in macrophages, with help from complement C3, providing a potential pathway for HFD-induced CVDs (50). Indeed we noticed that almost all complement components are upregulated upon HFD, driving the forward cascade, while negative regulators, such as *Serpin1b*, were largely downregulated. The crescendo forward signaling and suppressed negative feedback initiate chronic inflammatory response, leading to detrimental outcome, even diseases. In this regard, it is similar to the cardiac resident macrophages from aging mice (51), accentuating the tight links among metabolism, inflammation, and aging.

The potential mechanism underlying lipid metabolism and inflammation has long been studied—for example, mitochondrion complex I deficiency caused glucose metabolism pattern shift and mitochondrial ROS, thus impairing macrophage efferocytosis (52). The mitochondrion–ER interface was also identified to promote NLRP3 inflammasome activation and IL-1β production (53). However, how other organelles work during the crosstalk between lipid metabolism and inflammation has not been well established. In the current study, we found a series of DEGs encoding proteins in Golgi apparatus and ER in CRM from HFD-fed mice, such as *Tlr6* and *Apod* in Golgi apparatus. TLR6 has been shown to contribute to atherosclerosis by a specific agonist in HFD-fed *Ldlr* ^-/-^ mice (54), while our study demonstrates that HFD feeding could activate cardiac resident macrophages and contribute to inflammation. Another example is ApoD, a transporter of several small hydrophobic molecules. Although mostly associated to lipid metabolism, ApoD is barely expressed in liver and intestines, the organs that normally produce apolipoproteins (55). ApoD has also been shown to dampen inflammation in adipose, probably through binding with osteopontin, while our data showed that ApoD in Golgi apparatus could promote inflammation in cardiac macrophages, and revealing how ApoD works in Golgi apparatus would extend our knowledge on its function and regulatory mechanisms of inflammation in cardiac macrophages.

There were still some limitations in the current study. First, only female mice were used, which could lead to a potential sex bias, given sex-related differences in metabolism and immune response. Second, we observed that the number of CCR2^-^ macrophages was elevated in HFD-fed mice’s hearts, and we are not sure if it is a general trend for HFD feeding or just a time section observation. As cardiac resident macrophages are essential for cardiac homeostasis, it is possible that resident macrophages experience self-renewal upon high fat stress (56) to maintain cardiac homeostasis, which surely deserves further research. Bone marrow transplant or heart transplant may be helpful to resolve this issue.

This study provides a general scenario of DEGs in cardiac resident macrophages upon HFD. Validation and screening of more functional genes/signaling pathways surely will shed more light on its role during the processes of metabolism, inflammation, and cardiovascular diseases, even aging. Although our study only drew up an interesting outline about resident macrophage gene expression change, further research is needed to explore how to modify cardiac resident macrophages to benefit cardiovascular disorders.

## Data availability statement

The original contributions presented in the study are publicly available. This data can be found here: GEO - GSE253651.

## Ethics statement

The animal study was approved by Institute of Animal Care and Use Committees. The study was conducted in accordance with the local legislation and institutional requirements.

## Author contributions

SZ: Data curation, Investigation, Methodology, Software, Supervision, Writing – original draft, Writing – review & editing. YL: Data curation, Formal analysis, Investigation, Methodology, Writing – review & editing. GX: Investigation, Software, Writing – original draft. XW: Formal analysis, Writing – review & editing. AD: Methodology, Writing – review & editing. JW: Formal analysis, Writing – review & editing. YpW: Formal analysis, Writing – review & editing. YlW: Writing – review & editing. CS: Supervision, Writing – review & editing. PW: Supervision, Writing – review & editing. CX: Conceptualization, Formal analysis, Funding acquisition, Supervision, Writing – review & editing, Writing – original draft.
