# Supplementary material for: Modulation of cardiac resident macrophages immunometabolism upon high-fat-diet feeding in mice
Source: Front Immunol. 2024 Jun 28;15:1371477. doi: 10.3389/fimmu.2024.1371477 (PMC11239335; doi:10.3389/fimmu.2024.1371477)
Supplement: Supplementary file 1 [file DataSheet_1.docx]

Supplementary Material

Modulation of cardiac resident macrophages immunometabolism upon high-fat diet feeding in mice

Simeng Zhu^1, *^, Yujia Liu^2, *^, Guofang Xia^1^, Xiaoqing Wang^1^, Ailian Du^2^, Jin Wu^3^, Yanpeng Wang^1^, Chengxing Shen^1^, Yuanlong Wang^1, †^, Peng Wei^1, †^, Congfeng Xu^1, †^

# Supplementary Figures and Tables.

## Supplementary Table

| Table s1. Primers for RT-PCR. | | |
| --- | --- | --- |
| Gene | Forward (5' to 3') | Reverse (3' to 5') |
| Apod | TCACCACAGCCAAAGGACAAA | CGTTCTCCATCAGCGAGTAGT |
| Cx3cl1 | ACGAAATGCGAAATCATGTGC | CTGTGTCGTCTCCAGGACAA |
| Tlr6 | TGAGCCAAGACAGAAAACCCA | GGGACATGAGTAAGGTTCCTGTT |
| Kars | CGCCGGGGAGACATAATTGG | TCTTTGACGATACCGTGTTTCC |
| Ahsg | GCTGCCACTGACTGTACTG | CTGTGGGTACGGGACCTAC |
| Ptpn1 | GGAACTGGGCGGCTATTTACC | CAAAAGGGCTGACATCTCGGT |
| Aoc3 | GAAGACCACCCTAGTGCTCCT | ATGAAGAGGTTGGCTCAGTCC |
| Atp7a | TGGGAAAGTGAATGGTGTCCA | ACGGTATTGGTTAAGACAGGGA |

## Supplementary Figures

**Figure s1.**


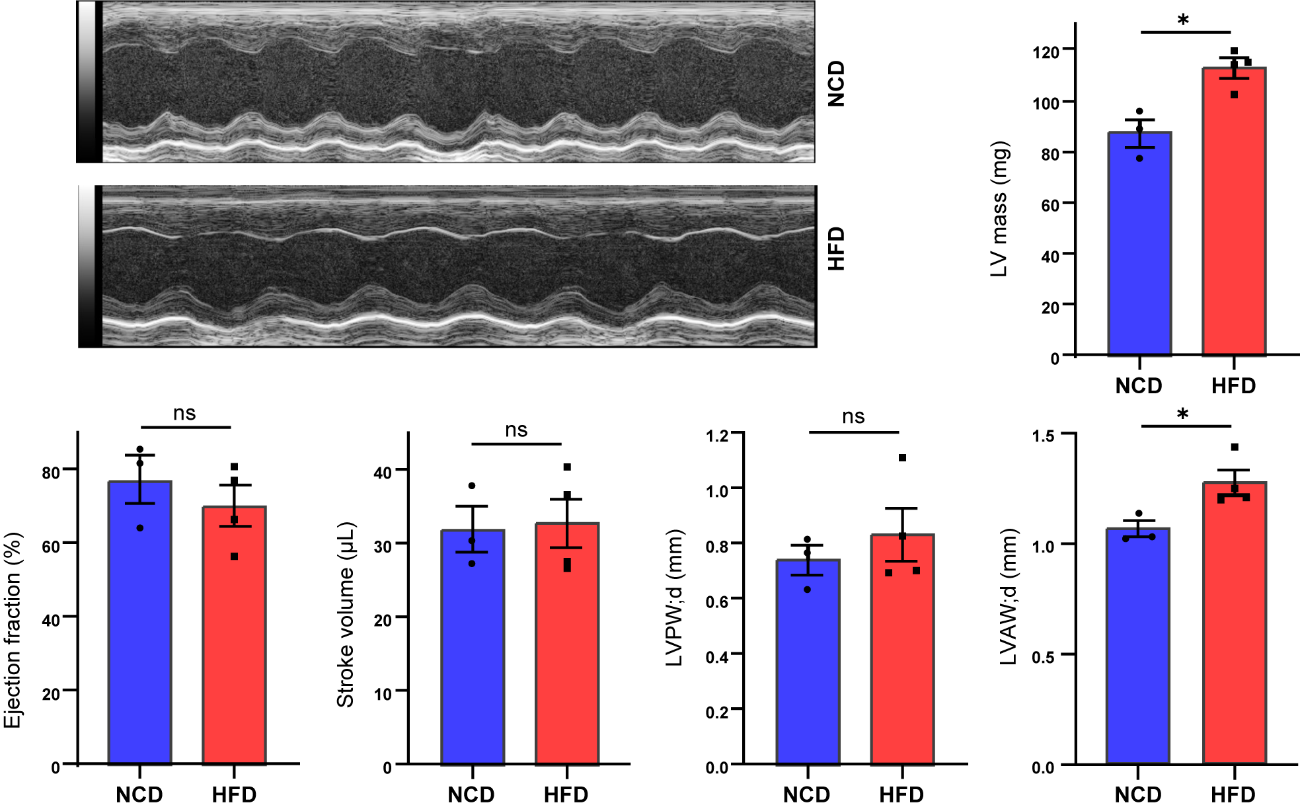


**Figure s1. Cardiac systolic function alterations in HFD fed mice.** Systolic function of NCD and HFD fed mice measured by echocardiography.

**Figure s2.**


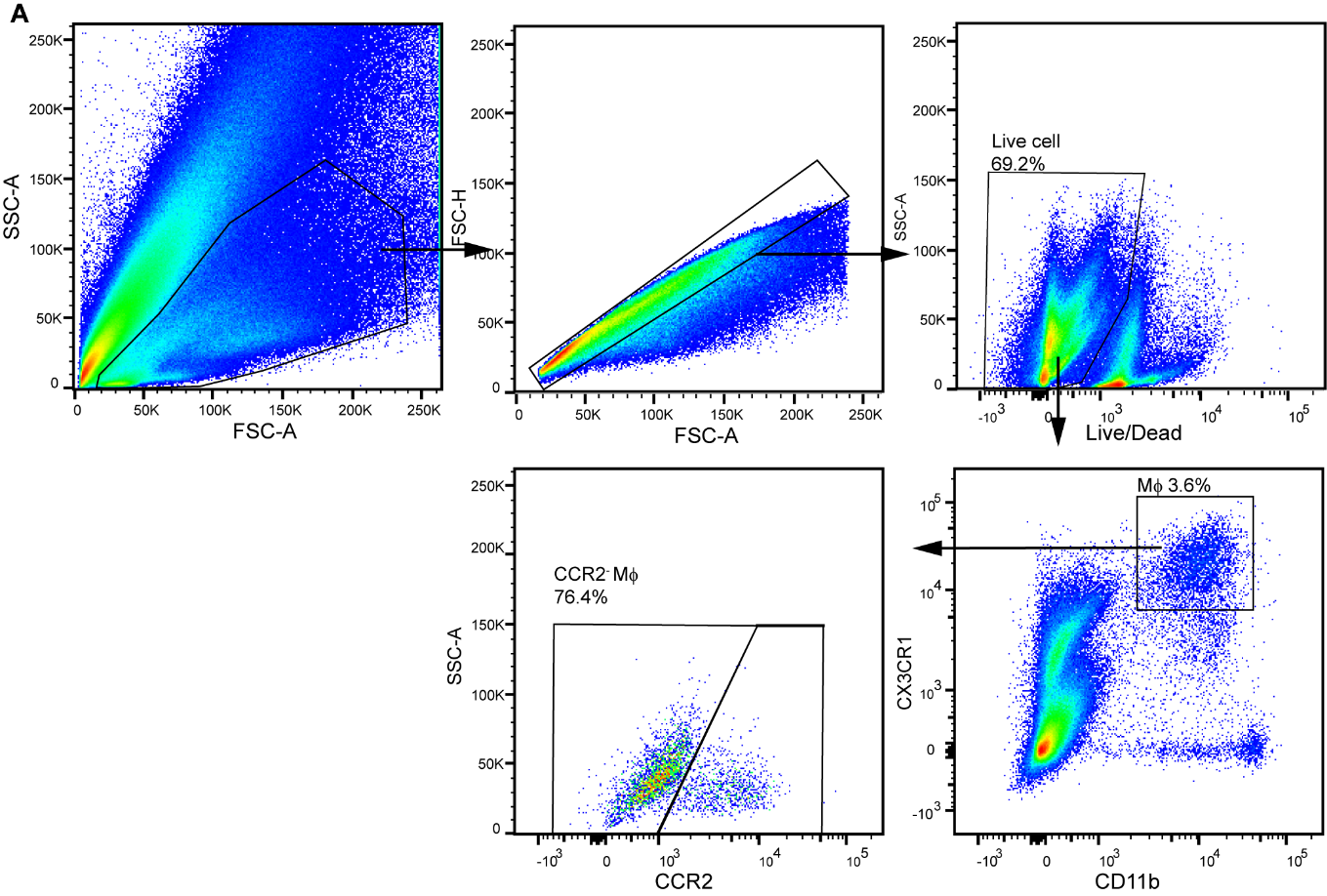


**Figure s2. Sorting strategies for cardiac macrophages.** Cardiac resident macrophages were sorted by flowcytometry with the indicated markers.

**Figure s3.**


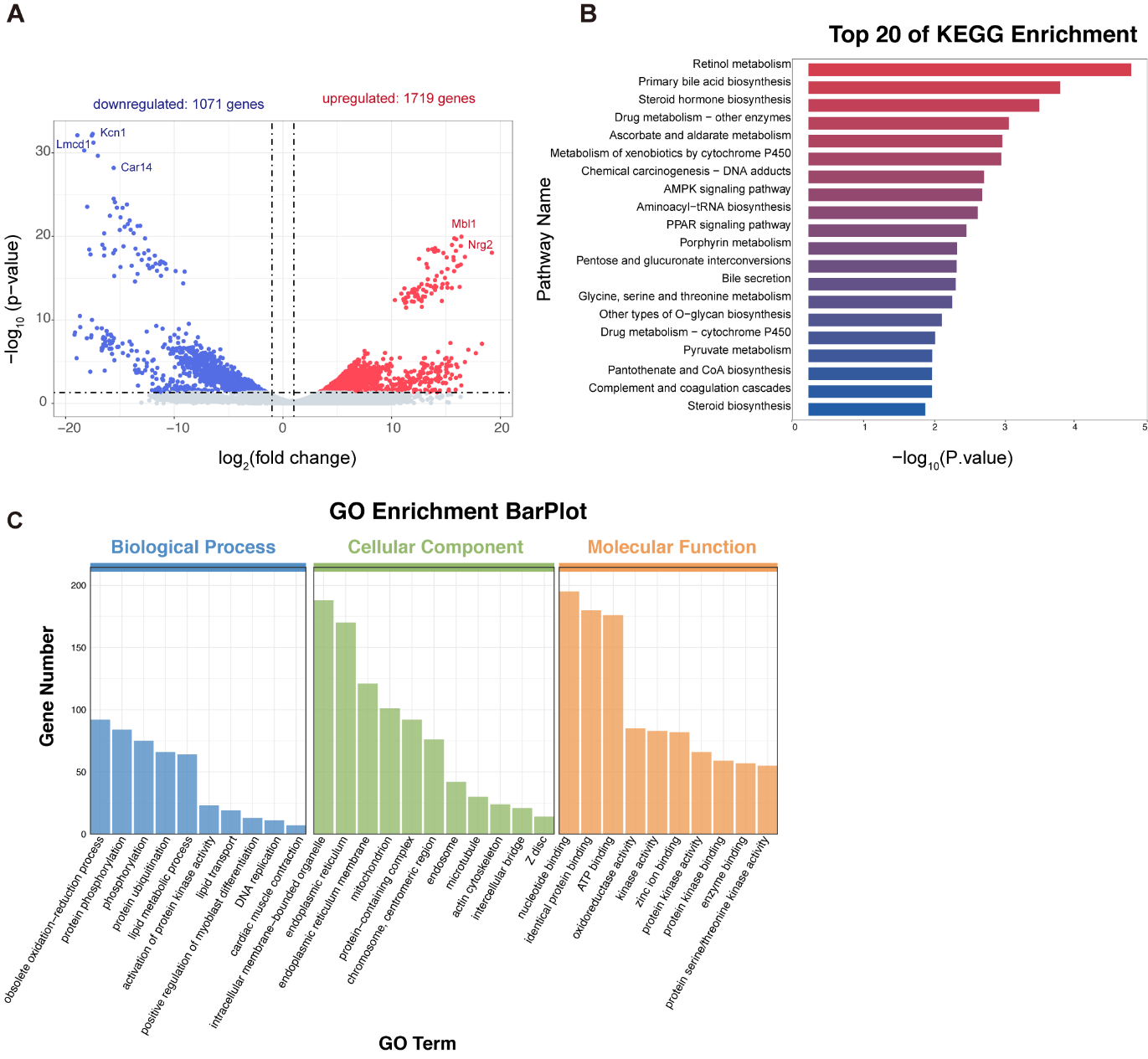


**Figure s3. Smart RNA sequencing for CCR2+ macrophages.** The DEGs in CCR2+ macrophages were plotted as volcano plot (A) and enriched by KEGG (B) and GO analysis (C).
